# Treatment and Controversies in Paraesophageal Hernia Repair

**DOI:** 10.3389/fsurg.2015.00013

**Published:** 2015-04-20

**Authors:** Abraham Lebenthal, Stephen D. Waterford, P. Marco Fisichella

**Affiliations:** ^1^Department of Surgery, Brigham and Women’s Hospital, Boston VA Health Care System, Boston, MA, USA; ^2^Division of Thoracic Surgery, Brigham and Women’s Hospital, Boston VA Health Care System, Boston, MA, USA; ^3^Department of General Surgery, Massachusetts General Hospital, Boston, MA, USA

**Keywords:** hiatal hernias, paraesophageal hernias, gastroesophageal reflux disease, iron-deficiency anemia, mesh repair

## Abstract

**Background:**

Historically all paraesophageal hernias were repaired surgically, today intervention is reserved for symptomatic paraesophageal hernias. In this review, we describe the indications for repair and explore the controversies in paraesophageal hernia repair, which include a comparison of open to laparoscopic paraesophageal hernia repair, the necessity of complete sac excision, the routine performance of fundoplication, and the use of mesh for hernia repair.

**Methods:**

We searched Pubmed for papers published between 1980 and 2015 using the following keywords: hiatal hernias, paraesophageal hernias, regurgitation, dysphagia, gastroesophageal reflux disease, aspiration, GERD, endoscopy, manometry, pH monitoring, proton pump inhibitors, anemia, iron-deficiency anemia, Nissen fundoplication, sac excision, mesh, and mesh repair.

**Results:**

Indications for paraesophageal hernia repair have changed, and currently symptomatic paraesophageal hernias are recommended for repair. In addition, it is important not to overlook iron-deficiency anemia and pulmonary complaints, which tend to improve with repair. Current practice favors a laparoscopic approach, complete sac excision, primary crural repair with or without use of mesh, and a routine fundoplication.

## Introduction

Paraesophageal hernia comprises 5% of all hiatal hernias. While historically all paraesophageal hernias were surgically repaired, intervention is now reserved for symptomatic paraesophageal hernias. In this review, we describe the indications for repair of paraesophageal hernia repair. Next we explore the controversies in paraesophageal hernia repair, which include a comparison of open to laparoscopic paraesophageal hernia repair, the necessity of complete sac excision, the routine performance of fundoplication, and the use of mesh for hernia repair.

## Methods

We searched Pubmed for papers published between 1980 and 2015 using the following keywords: hiatal hernias, paraesophageal hernias, regurgitation, dysphagia, gastroesophageal reflux disease, aspiration, GERD, endoscopy, manometry, pH monitoring, proton pump inhibitors, anemia, iron-deficiency anemia, Nissen fundoplication, sac excision, mesh, and mesh repair. We found a total of 5743 papers. As we were not performing a meta-analysis of all clinical results in paraesophageal hernia, but rather providing an experience-based review of the most impactful contributions to the literature, we selected 36 papers for inclusion in our review. These represent substantial contributions to the field of paraesophageal hernia repair.

## Incidence and Clinical Presentation

Paraesophageal hernia presents at a median age of 65–75 years, based on several large series in the literature (1–3). It is believed that most patients with paraesophageal hernia are asymptomatic. Symptoms can arise from obstruction, reflux, or bleeding. Obstruction at the gastroesophageal junction (GEJ) or at the level of the pylorus can occur from intermittent twisting of the stomach along its long axis while herniating into the chest. If the GEJ is obstructed, the patient will complain of dysphagia and regurgitation, while gastric outlet obstruction produces nausea, vomiting, and epigastric or chest pain. Gastroesophageal reflux disease (GERD) is more common in sliding hiatal hernia, but can occur in paraesophageal hernia as well. In a series of 95 consecutive patients with GERD, those with a sliding hiatal hernia over 3 cm had a significantly shorter lower esophageal sphincter (LES) and greater reflux on pH monitoring compared to those with no sliding hiatal hernia or a sliding hiatal hernia <3 cm (4). Bleeding from the herniated fundus of the stomach owing to mucosal ulcers, known as Cameron lesions, can produce iron-deficiency anemia. Regardless of mechanism, many patients with paraesophageal hernia have other non-specific symptoms, such as postprandial chest pain, postprandial fullness, and shortness of breath. Finally, patients can present acutely with strangulation of the stomach from acute gastric volvulus, which constitutes a surgical emergency. These patients retch but cannot vomit, and a nasogastric tube cannot be passed into the stomach (5).

## Diagnosis

**Figure 1:**
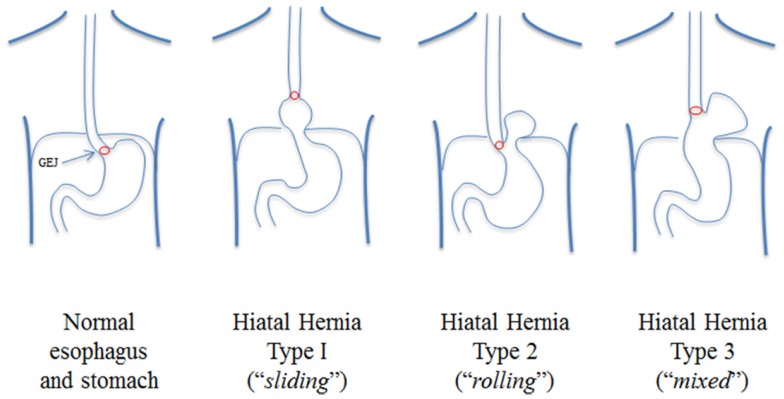
**Classification of hiatal hernias: paraesophageal hernias are of type III from Ref. (5)**.

An essential diagnostic test for paraesophageal hernia is a barium swallow, which demonstrates the amount and position of stomach within the thorax. We have found these images to be critical because they demonstrate the location of the GEJ, distinguishing a type II from a type III paraesophageal hernia (5). Hiatal hernias are classified into four types (5) and type III, known as a “mixed” paraesophageal hernia, is a true paraesophageal hernia and results from a combination of sliding type I and rolling type II hernia, with the stomach migrated into the chest and “rolled” over the stomach, with concomitant migration of the GEJ into the chest (Figure 1). In the evaluation of paraesophageal hernia, upper endoscopy is performed to demonstrate the presence of mucosal lesions, as well as to determine whether esophagitis and Barrett’s esophagus are present. Finally, esophageal manometry is used to assess esophageal motility, which influences selection of the type of fundoplication (partial or total). Placement of a manometry catheter can be difficult in the setting of paraesophageal hernia, and can be guided by endoscopy if necessary. Esophageal pH monitoring is usually performed in the presence of GERD symptoms to document the presence of abnormal esophageal acid exposure. However, if a patient has dysphagia, no pH monitoring is performed, as dysphagia alone suffices as an indication for surgery and pH monitoring would not later the treatment algorithm.

## Treatment

Traditionally, all paraesophageal hernias were recommended for repair to prevent strangulation of hernia contents and to avoid the mortality of emergent repair, but this recommendation has changed as an appreciation for the morbidity and mortality of elective repair has increased (5). The analysis of the nationwide inpatient sample (NIS) in 1997 revealed that the mortality of emergency surgery was lower than expected, at 5.4%, and that the annual probability of requiring emergency surgery during watchful waiting of a paraesophageal hernia was 1.1% (6). This contrasted with a mortality rate for elective repair of 1.4%. These findings suggested that watchful waiting was an appropriate strategy for asymptomatic or minimally symptomatic paraesophageal hernias. Minimally symptomatic hernias were defined as those that did not affect the quality of life of the patient, and included symptoms such as belching and heartburn. Conversely, symptomatic paraesophageal hernias were recommended for repair.

The major issue in clinical decision-making in paraesophageal hernia concerns the assessment of symptoms. Some studies have suggested that over half of patients with paraesophageal hernias are asymptomatic (7), but the true number remains unknown owing to lack of population studies. Carrott and colleagues suggested that symptoms associated with paraesophageal hernia are much broader than previously suggested, and that truly asymptomatic patients are, in fact, rare (8). They also describe that type of symptoms correlates with the anatomy of the hernia. In this single-center review of 270 consecutive patients undergoing surgical repair of paraesophageal hernia, they found that symptoms were wide-ranging, and included heartburn (65%), early satiety (50%), chest pain (48%), dyspnea (48%), dysphagia (48%), and regurgitation (47%). In addition, anemia was present in 41%. Specifically, 269 of 270 patients in this series were symptomatic, and the median number of symptoms was 4. At a median post-operative follow-up of 103 days, symptoms improved in patients with heartburn (93%), early satiety (79%), chest pain (76%), dyspnea (67%), dysphagia (81%), and regurgitation (92%). This study was limited because all patients underwent surgery for symptomatic hernia, and therefore a population of incident paraesophageal hernias was not available to determine whether they were truly asymptomatic (8). Nonetheless, the authors suggest that patients with paraesophageal hernias are often labeled as asymptomatic or minimally symptomatic, because the hernia has been present for years in an older patient and the gradual alterations in eating and postprandial symptoms are attributed to aging. Moreover, the symptoms specific to larger paraesophageal hernia, such as dysphagia, early satiety, and positional dyspnea, are often insidious and increase only over the course of years. Carrott and colleagues also suggested that a surgeon experienced in repair should evaluate all surgically fit patients with paraesophageal hernia, as surgical mortality was 0 in their series.

While gastrointestinal symptoms of paraesophageal hernia are the main focus of indications for repair, pulmonary symptoms represent an underappreciated symptom of paraesophageal hernia. In fact, many paraesophageal hernia repair series in the literature do not assess patients for dyspnea, likely because in this elderly population dyspnea is often assumed to arise from other comorbidities (9). The benefits of paraesophageal repair in patients with respiratory complaints have been studied. In a series of 120 patients who had pulmonary function tests (PFTs) before and after repair of a giant paraesophageal hernia, 52% complained of dyspnea preoperatively (9). There was a mean change of 10.3% in percent predicted forced expiratory volume in 1 s (FEV_1_) after repair in the group as a whole, and 75% of patients complaining of dyspnea described complete relief with repair (9). PFTs improved the most in patients with the greatest amount of intrathoracic stomach. The mechanism of pulmonary impairment in paraesophageal hernia likely involves reduction in thoracic volume, as well as the stomach being drawn into the chest during inspiration by negative intrapleural pressure, indicating that the hernia contents behave as an internal flail segment (9). These results demonstrate that symptom questionnaires for patients with paraesophageal hernia should include respiratory symptoms, and that dyspnea should be considered a symptom of paraesophageal hernia, which can be improved by operative intervention.

Less appreciated is the possibility that a large paraesophageal hernia can compress the heart, producing exertional dyspnea through a mechanism other than lung compression or diaphragm dysfunction. In a study of 30 patients with paraesophageal hernia who had normal PFTs preoperatively with a mean FEV_1_ of 99% predicted, 25 complained of exertional dyspnea (10). The authors performed resting and stress echocardiography and cardiac computed tomography (CT) in all 30 patients, and found that 23 (77%) had moderate to severe compression of the left atrium, 11 (37%) had right inferior pulmonary vein compression, 12 (40%) had left inferior pulmonary vein compression, and 26 (87%) had coronary sinus compression on cardiac CT. In patients with severe left atrial compression, there was a significant increase in left ventricular end-diastolic and end-systolic volumes after repair on echocardiography, and left atrial volume increased significantly after repair. Finally, most patients in the study were New York Heart Association (NYHA) functional class II and III preoperatively, and most improved to NYHA class I post-operatively. The identification of left atrial compression preoperatively may identify a group of patients likely to benefit from paraesophageal hernia repair.

Among other presenting symptoms, iron-deficiency anemia may associate with a paraesophageal hernia. The prevalence of patients with paraesophageal hernia who have iron-deficiency anemia has been investigated extensively. Segal reported that hiatal hernia was associated with anemia in 1931 (11), and Bock and colleagues reported a series of 10 patients in the New England Journal of Medicine in 1933 who had diaphragmatic hernia and anemia (12). It was speculated that venous congestion and arterial obstruction within the herniated stomach was the source of bleeding. Collis himself in 1967 described 400 patients with hiatal hernia of whom 15% were anemic (13). He demonstrated that 37 of 326 patients with sliding hiatal hernia (11%) were anemic, but that 22 of 74 patients with paraesophageal hernia (30%) were anemic. Further, on esophagoscopy, he found a low incidence of reflux esophagitis in paraesophageal hernias, suggesting that GERD was not the cause of anemia. Operative repair of the hiatal hernias was associated with a mean rise in Hg of 5.4 g/dL in the anemic patients undergoing repair. In 1986, Cameron described a series of 109 patients with large paraesophageal hernia, defined as intrathoracic presence of one-third of the stomach, 55 of whom had anemia and 54 of whom did not (14). Similar to Collis, Cameron found that the incidence of GERD and peptic ulcer did not differ between the groups, but that linear gastric erosions near the diaphragmatic hiatus were found in 23 (42%) of the anemic patients and 13 (24%) of the non-anemic patients. These are now termed “Cameron’s lesions.” More recent reports confirm a high incidence of Cameron lesions and iron-deficiency anemia. In a series of 77 patients with anemia who underwent repair of giant paraesophageal hernia, defined in this series as a hernia involving over half of the stomach, 32% had Cameron lesions, and hemoglobin levels rose from a mean preoperative level of 9.6–13.2 mg/dL at 3- to 12-month follow-up and 13.6 mg/dL at 1 year follow-up (15). In another series of 183 patients undergoing paraesophageal hernia repair, 37% were anemic and 57% of the anemic patients were symptomatic from anemia or specifically referred for repair owing to anemia (16). At follow-up, 60% of patients had resolution of anemia: 70% in the symptomatic group and 48% in the asymptomatic group. In a subset of patients with Cameron lesions found on preoperative endoscopy, 88% had resolution of anemia, although 50% of patients without visible Cameron lesions also had resolution of anemia, suggesting that some patients without Cameron lesions had bleeding related to the hernia not present at the time of endoscopy. Overall, these studies suggest that anemia is common in patients with paraesophageal hernia, and routine investigation for iron-deficiency anemia in patients with paraesophageal hernia is warranted.

Patients younger than 65 years with minimal comorbidities and asymptomatic paraesophageal hernias are often recommended for repair, given the low morbidity of the operation in these patients (17). Finally, many patients with paraesophageal hernia are morbidly obese, given that increased abdominal pressure predisposes to paraesophageal hernia, and these patients should be considered for combined bariatric surgery and paraesophageal hernia repair. This approach reduces risk for recurrent paraesophageal hernia, which is increased by obesity, and addresses morbid obesity at the same operation (18). Patients can undergo either sleeve gastrectomy or gastric bypass concurrent with paraesophageal hernia repair, although those who experience severe GERD should preferentially undergo gastric bypass, as sleeve gastrectomy does not eliminate GERD and may actually worsen it. In a retrospective review of 4832 patients who underwent laparoscopic sleeve gastrectomy, 44.5% of patients had GERD preoperatively, and of those with preoperative GERD, 84.1% of patients continued to have GERD symptoms, with only 15.9% demonstrating resolution (19). Among the laparoscopic sleeve gastrectomy patients who did not have GERD preoperatively, 8.6% developed it post-operatively. The authors suggested that GERD might represent a relative contraindication to sleeve gastrectomy.

## Controversies in Paraesophageal Hernia Repair

### Laparoscopic versus open repair

Laparoscopic paraesophageal hernia repair has recently gained popularity. However, a recent analysis of the NIS from 1999 to 2008 indicates that 91% of paraesophageal hernia repairs were performed open (74.4% open abdominal, 17% thoracotomy), while 9% were performed laparoscopically (20). The authors found that while mortality was similar, patients who underwent open hernia repairs had longer length of stay. A review of the literature has also demonstrated that while there are no randomized trials to compare laparoscopic and open repair, laparoscopic repair may also be associated with fewer complications, such as pneumonia, thrombosis, hemorrhage, and urinary tract and wound infections (21). Several series of laparoscopic repairs of giant paraesophageal hernia have also reported excellent outcomes (1). Nevertheless, concern persists over hernia recurrence after laparoscopic repair. In one series of 60 paraesophageal hernia repairs, recurrence rates of 44% were reported for laparoscopic repair versus 23% for open repair, although this was not statistically significant (22). Another report indicated that recurrence rate for laparoscopic repair after introduction of laparoscopic repair was 44 versus 15% for open repair, but that with experience the laparoscopic recurrence rate reached that observed after open repair (23). Optimizing technical performance of the repair may allow for laparoscopic repair to be offered to the majority of patients, providing the benefits of reduced morbidity with acceptable recurrence rates.

### Thoracic versus abdominal approach

Data from the NIS indicate that thoracotomy is associated with prolonged hospital stay, reduced discharge to home, greater need for post-operative mechanical ventilation, and increased rate of pulmonary embolism (20). The thoracic approach was used because it provides a direct approach to the hernia sac, ability to mobilize the esophagus farther superiorly, easy performance of Collis gastroplasty, and ready access for a relaxing incision in the left hemidiaphragm. In a series of 94 patients with massive, incarcerated paraesophageal hernias who underwent thoracotomy with fundoplication with median follow-up of 72 months, only 2 patients required re-operation for recurrent hernia (24). Nonetheless, the average hospital stay of 7.8 days in the NIS has resulted in a trend away from thoracotomy for repair of paraesophageal hernia (20). Today, the abdominal laparoscopic approach still remains the standard treatment.

### Sac excision

**Figure 2:**
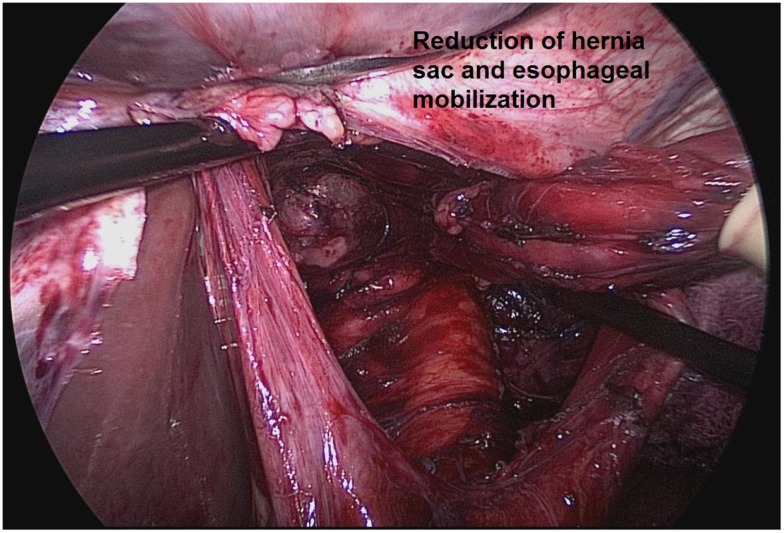
**Complete sac excision and mobilization of an adequate segment of intra-abdominal esophagus from Ref. (27)**.

There has been debate over the necessity of a complete sac excision. Some have suggested that partial sac excision, particularly when the sac is thick or densely adherent to mediastinal structures, is sufficient. However, it appears that complete sac excision is a factor that leads to decreased recurrence (25). One group reported that complete sac excision reduced early recurrence of paraesophageal hernia (25). Another series of 86 patients undergoing laparoscopic hiatal hernia repair compared a strategy of leaving the sac in the mediastinum to a strategy of primary dissection of the sac prior to esophageal mobilization. Conversion to open was required in 40% of the patients managed with the sac left in the mediastinum versus 9% of patients managed with complete sac dissection (26). The likely explanation for superior outcomes with complete sac excision relate to the ability to mobilize an adequate segment of intra-abdominal esophagus following sac excision (Figure 2).

### Antireflux procedure

Fundoplication is a standard component of paraesophageal hernia repair. A 20-year retrospective study of 95 paraesophageal hernia repairs without fundoplication published in 1973 indicated that there was a radiological recurrence rate of 33% following repair (28). As a result, fundoplication was included in paraesophageal hernia repair. A recent study examined this issue in 60 patients who underwent paraesophageal hernia repair, 35 of whom had repair with fundoplication and 25 of whom had repair without fundoplication (29). All patients with preoperative GERD underwent fundoplication. In the 25 patients who did not undergo fundoplication, there was a 28% incidence of esophagitis and a 39% incidence of abnormal esophageal acid exposure. The authors suggested that fundoplication should be a routine part of paraesophageal hernia repair. A study of 4 patients with type II paraesophageal hernia and 11 patients with type III paraesophageal hernia, all of whom underwent repair with fundoplication, demonstrated that at 1 year all patients were asymptomatic without dysphagia or reflux. The authors suggested that fundoplication restores competency to the LES and prevents post-operative reflux that would otherwise result from the extensive dissection required for paraesophageal hernia repair. Fundoplication may also help to anchor the stomach below the diaphragm, preventing recurrence (30).

### Use of mesh

The use of mesh during paraesophageal hernia repair has been an area of controversy. The high recurrence rates seen with repair of paraesophageal hernia, particularly those in laparoscopic series, led to an interest in use of mesh to reduce recurrence rates even though many recurrences of paraesophageal hernia are asymptomatic (28). In one series, there was an asymptomatic radiographic recurrence rate of 21% following laparoscopic repair, and a 12% rate of symptomatic recurrence requiring re-operation following laparoscopic repair (31). Similarly, in a series of 85 laparoscopic paraesophageal hernia repairs, only 1 patient required re-operation for symptomatic recurrence, while 23 of 35 (66%) of patients who underwent barium swallow at a median of 99 months had radiographic recurrence (32). Another series demonstrated a 42% recurrence rate after laparoscopic repair of type III hiatal hernias, but most were asymptomatic (33).

The use of mesh has been demonstrated to lower recurrence rates after laparoscopic paraesophageal hernia repair compared to cruroplasty alone. A prospective, randomized controlled trial of Nissen fundoplication with posterior cruroplasty versus Nissen fundoplication with posterior cruroplasty and onlay polytetrafluoroethylene (PTFE) mesh in 72 patients demonstrated a 22% recurrence rate in the cruroplasty alone group and a no recurrence in the mesh group (34). The remainder of the data in this area comes from observational studies. A review of several studies in the literature concluded that prosthetic mesh reduced paraesophageal hernia recurrence after laparoscopic repair (35). However, the use of prosthetic mesh has been associated with severe complications, including erosion into the esophagus and esophageal stenosis (36). Mesh infections can pose unique management problems. In some patients, gastrectomy can be required to remove mesh, which has eroded into the esophagus, or gastric cardia (36). Dysphagia owing to esophageal stenosis also requires re-operation for mesh removal.

Biologic mesh has been studied to determine whether it can reduce recurrence rate after paraesophageal hernia repair without causing the severe complications of permanent prosthetic meshes. A multicenter randomized controlled trial of primary repair versus biologic prosthesis consisting of small intestine submucosa randomized 108 patients undergoing laparoscopic paraesophageal hernia repair to one of the two groups (37). While the short-term recurrence rate was more than doubled at 6 months in the primary repair group, at a median follow-up of 58 months, 59% of patients in the primary repair group had a recurrence versus 54% in the biologic mesh group. There was also no statistically significant difference in quality of life amongst the two groups. As a result, owing to the asymptomatic nature of most recurrences, a properly performed cruroplasty may be appropriate choice for repair of paraesophageal hernias, without incurring the cost and potential side effects of use of mesh.

## Conclusion

Indications for paraesophageal hernia repair have changed, and currently symptomatic paraesophageal hernias are recommended for repair. However, given a lack of population-based data, it is difficult to determine the percentage of patients with paraesophageal hernias who are symptomatic, and many authors believe that most patients with paraesophageal hernia are symptomatic when questioned carefully. Many patients have slow-onset dysphagia or dyspnea over several years, which are relieved by hernia repair. In addition, it is important not to overlook iron-deficiency anemia and pulmonary complaints, which tend to improve with repair. Over recent decades, laparoscopic paraesophageal hernia repair has become the operation of choice compared to open repair, given lower rates of morbidity and shorter duration of hospital stay. There are several controversies in paraesophageal hernia repair; current practice favors a laparoscopic approach, complete sac excision, primary crural repair with or without use of mesh, and routine fundoplication. There are a limited number of randomized controlled trials in the field of paraesophageal hernia, and further studies in paraesophageal hernia will clarify these areas of controversy and guide the future management.

## Conflict of Interest Statement

The authors declare that the research was conducted in the absence of any commercial or financial relationships that could be construed as a potential conflict of interest.
